# Experimental Mixed-Gas Permeability, Sorption and Diffusion of CO_2_-CH_4_ Mixtures in 6FDA-mPDA Polyimide Membrane: Unveiling the Effect of Competitive Sorption on Permeability Selectivity

**DOI:** 10.3390/membranes9010010

**Published:** 2019-01-08

**Authors:** Giuseppe Genduso, Bader S. Ghanem, Ingo Pinnau

**Affiliations:** Functional Polymer Membranes Group, Advanced Membranes and Porous Materials Center, Division of Physical Science and Engineering, King Abdullah University of Science and Technology, Thuwal 23955-6900, Saudi Arabia; bader.ghanem@kaust.edu.sa

**Keywords:** mixed-gas sorption, mixed-gas diffusion, mixed-gas permeation, competitive sorption, gas separation membranes, 6FDA-mPDA polyimide

## Abstract

The nonideal behavior of polymeric membranes during separation of gas mixtures can be quantified via the solution-diffusion theory from experimental mixed-gas solubility and permeability coefficients. In this study, CO_2_-CH_4_ mixtures were sorbed at 35 °C in 4,4′-(hexafluoroisopropylidene)diphthalic dianhydride (6FDA)-m-phenylenediamine (mPDA)—a polyimide of remarkable performance. The existence of a linear trend for all data of mixed-gas CO_2_ versus CH_4_ solubility coefficients—regardless of mixture concentration—was observed for 6FDA-mPDA and other polymeric films; the slope of this trend was identified as the ratio of gas solubilities at infinite dilution. The CO_2_/CH_4_ mixed-gas solubility selectivity of 6FDA-mPDA and previously reported polymers was higher than the equimolar pure-gas value and increased with pressure from the infinite dilution value. The analysis of CO_2_-CH_4_ mixed-gas concentration-averaged effective diffusion coefficients of equimolar feeds showed that CO_2_ diffusivity was not affected by CH_4_. Our data indicate that the decrease of CO_2_/CH_4_ mixed-gas diffusion, and permeability selectivity from the pure-gas values, resulted from an increase in the methane diffusion coefficient in mixtures. This effect was the result of an alteration of the size sieving properties of 6FDA-mPDA as a consequence of CO_2_ presence in the 6FDA-mPDA film matrix.

## 1. Introduction

Conventional distillation or absorption and adsorption systems are reliable and, for existing plants, economically feasible technologies. However, membrane units could potentially replace these traditional unit operations, ensuring better economics and lower environmental impact [1]. To fully exploit the potential of membrane-based gas-separations, polymeric materials of high permeability and permeability selectivity are required. Moreover, these materials must be mechanically strong for formation of integral asymmetric and thin-film composite membranes and should also ensure stable separation performances over time. Currently, most experimental studies reported in the literature are based on pure-gas permeation properties of membrane materials (e.g., cellulose acetate, polysulfone, polyphenylene oxide, polyimides, polymers of intrinsic microporosity (PIM), and others) [2,3,4,5,6]. However, when gas permeation is performed under mixed-gas conditions, permeability and permeability selectivity can deviate significantly from the pure-gas values. For example, the pure-gas CO_2_/CH_4_ permeability selectivity of PIM-1 is ~15 (in the range of 0–10 atm CO_2_ pressure), but drops to ~8 in a CO_2_/CH_4_ mixture at 10 atm partial CO_2_ pressure [4], that is, only ~50% of the ideal value. Because the overall intention of CO_2_-CH_4_ separation studies is to design membrane materials for industrial applications, correct identification of the reasons for the deviation from ideality of permeability selectivity is crucial. Therefore, one should describe gas transport at both thermodynamic (sorption) and kinetic (diffusion) levels. The solution-diffusion theory that is commonly applied to describe the transport of fluids through dense polymer membranes affirms that permeability (*P_i_*) can be directly determined as the combination of solubility (*S_i_*) and diffusion (*D_i_*) coefficients of a specific gas in a membrane [7]. Hence, to accurately describe the transport of gases through membranes, one should preferably couple mixed-gas permeation data, measured by a well-established experimental technique [8], with mixed-gas sorption or diffusion data. Fraga et al. [9], for example, designed and implemented a new time lag apparatus for the direct measurement of mixed-gas permeability and diffusion coefficients in Pebax 2533, HyflonAD60X, and PIM-EA-TB—the same instrument was used by Monteleone et al. [10] to test mixed-gas diffusion coefficients of a spirobifluorene-based polymer of intrinsic microporosity (PIM-SBF-1). On the basis of our experimental capabilities, we estimated mixed-gas diffusion coefficient values by applying the solution-diffusion theory and combining experimental mixed-gas permeability and mixed-gas sorption values. Particularly, a newly developed sorption system was used for the mixed-gas sorption studies reported here [11]. To the best of our knowledge, so far, four types of mixed-gas sorption units have been described in the literature [11,12,13,14,15] and utilized to test a total of only 11 polymers [11,12,13,14,15,16,17,18,19,20,21,22,23]. Furthermore, the number of studies covering the diffusion of gas mixtures in polymers—retrieved from experiments of mixed-gas sorption and permeation—is even smaller. Some previous studies include the following: CO_2_-C_2_H_6_ in XLPEO [24], *n*-C_4_H_10_-CH_4_ in poly [1-trimethylsilyl-1-propyne] (PTMSP) [25], CO_2_-CH_4_ in 6FDA-TADPO [22], CO_2_-CH_4_ in PEO-based multi-block copolymer [26], *n*-C_4_H_10_-CH_4_ in polydimethylsiloxane (PDMS) [27], and CO_2_-CH_4_ in PDMS [11]. In our previous work [11], we showed that co-permeation of CO_2_ in mixtures with CH_4_ in rubbery polydimethylsiloxane (PDMS) increased mixed-gas diffusion of CH_4_.

**Figure 1:**
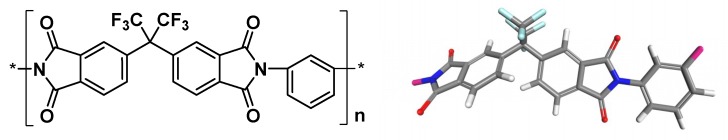
Chemical structure and 3D representation of the repeat unit of 4,4′-(hexafluoroisopropylidene)diphthalic dianhydride (6FDA)-m-phenylenediamine (mPDA) polyimide.

Similar to other commercial glassy polymers (e.g., polysulfones, cellulose acetates), polyimides have attracted the attention of the academic and industrial community for the following reasons: (i) strong thermal and mechanical properties (high glass transition and thermal decomposition temperatures, high ultimate tensile strength/elongation at break, and Young’s modulus) and (ii) excellent combination of pure-gas permeability and permeability selectivity [5]. In particular, polyimides based on the commercially available monomer 4,4′-(hexafluoroisopropylidene)diphthalic dianhydride (6FDA) are typically solution processable and, therefore, suitable for hollow fibers spinning [5,28]. The polycondensation reaction of 6FDA with m-phenylenediamine (mPDA)—a commercially available monomer—yields the high-performance 6FDA-mPDA polyimide [29,30], shown in Figure 1. 6FDA-mPDA displays an interesting combination of pure-gas CO_2_ permeability of ~14 Barrer and CO_2_/CH_4_ permeability selectivity of ~70 measured at 2 atm and 35 °C [27]. Therefore, this membrane material is a particularly attractive polymer to perform a case study of mixed-gas sorption and diffusion of CO_2_-CH_4_ mixtures in polyimides. Such a study may also clarify whether the deviation of the CO_2_ mixed-gas permeability from the pure-gas values can be ascribed to *competitive sorption* phenomena—as commonly assumed in the literature [31,32,33]—or to phenomena related to CO_2_-induced dilation [34].

In this work, we aimed to provide a full experimental description of sorption and diffusion of CO_2_-CH_4_ mixtures in isotropic 6FDA-mPDA films at 35 °C. First, we show the results of CO_2_-CH_4_ mixed-gas sorption experiments, and then perform an analysis of mixture effects on the solubility coefficients and solubility selectivity at various equilibrium pressures. To analyze mixed-gas solubility data, isothermal surfaces of gas uptake were estimated via the following: (i) linear interpolation and (ii) the extension of the dual-mode sorption model [35] to mixtures supported by an empirical expression for better data fitting. In the second part of this work, experimental mixed-gas permeability values of 6FDA-mPDA obtained with equimolar CO_2_-CH_4_ mixtures previously reported by our group [29] were divided by experimental mixed-gas solubilities to estimate CO_2_-CH_4_ mixed-gas concentration-averaged effective diffusion coefficient data. These data show the interaction between CO_2_ and the polymeric matrix of 6FDA-mPDA and the effect of CO_2_ diffusion on CH_4_ diffusion. Moreover, using our experimental data, we were able to clarify the impact of competitive sorption and CO_2_-sorption related phenomena on transport and separation of CO_2_-CH_4_ mixtures in 6FDA-mPDA.

## 2. Materials and Methods

### 2.1. Materials

6FDA-mPDA was synthesized according to the procedure reported elsewhere [29]. The polymer had a weight-averaged molecular weight (Mw) of 141,000 g/mol and a polydispersity index (Mw/Mn) of 1.2. Isotropic polyimide films made by solution casting from chloroform were air-dried, soaked in methanol for 12 h, and then dried at 120 °C under vacuum for 24 h. Complete solvent removal was confirmed by thermal gravimetric analysis (TGA). The geometric density of 6FDA-mPDA was determined at room temperature (22 °C) from membrane area (via image scanning), thickness (547-400S micrometer, Mitutoyo, Japan), and weight measurements (XPE204, Mettler Toledo, Columbia, SC, USA). Three pieces of 6FDA-mPDA film of 50 microns and three pieces of 240 microns were measured with fresh samples (i.e., 0 days aging). The geometric density of 6FDA-mPDA was 1.42 ± 0.02 g/cm^3^. Moreover, the density of 240-µm thick 6FDA-mPDA films aged for >3 months was identical to that of the fresh film samples. This value was slightly lower than other values reported in the literature—that is, 1.464 g/cm^3^ [36], 1.46 g/cm^3^ [37,38], 1.456 ± 0.014 g/cm^3^ [28], and 1.45 ± 0.01 g/cm^3^ [39], which were measured via the Archimedes’ principle procedure.

Certified gas mixtures of 11 mol% and 90 mol% CO_2_ in CH_4_ were purchased from Air Liquide; gas mixtures of 37 mol% and 51 mol% CO_2_ in CH_4_ were purchased from AHG Specialty Gas Center (Jeddah, Saudi Arabia).

### 2.2. Methods

#### 2.2.1. Barometric System

**Figure 2:**
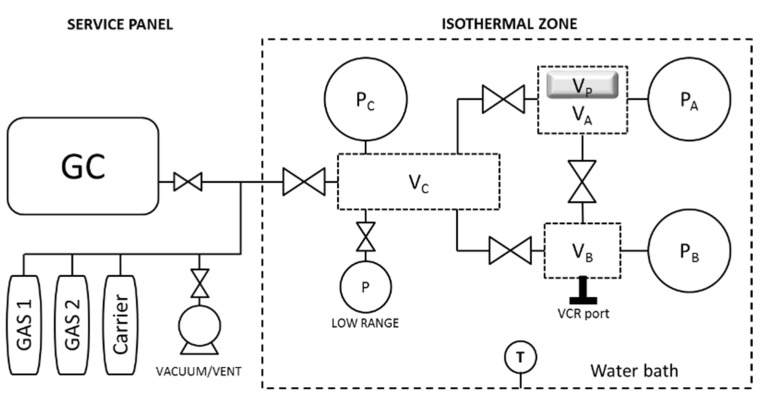
Schematic of the barometric mixed-gas unit used for sorption experiments discussed in this work (adapted from the literature [11]). GC—gas chromatograph.

The design and operation of the system used for barometric pure- and mixed-gas sorption experiments were introduced in detail elsewhere [11] and are shown here in Figure 2. In brief, gases are introduced from volume V_B_ to V_A_, which contains the polymer sample (V_P_). Volume V_C_ is connected to V_A_, V_B_, the gas chromatograph (GC, Agilent 490 Micro GC Natural Gas Analyzer, Santa Clara, USA), gas cylinders (custom-made mixtures and carrier gas), and a vacuum pump. V_C_ allows a certain operational flexibility to this mixed-gas system; for example, by addition from V_C_, gases can be mixed in V_B_. V_C_ can receive gas samples from both V_B_ and V_A_ for GC analysis. Furthermore, when using custom-made mixtures, the valve between V_B_ and V_C_ can be left open to increase the volume of the feed chamber so that more gas can be expanded to V_A_. P_A_ and P_B_ transducers of 35 atm range were used for both volumes V_A_ and V_B_ and were exchanged with transducers of 50 and 100 atm range, respectively, to explore high partial pressures (>15 atm partial gas pressure).

Active volumes in a barometric sorption system are the feed and the sample chamber because they are used to calculate gas uptakes by mass balance. Dead-volumes in tubes, transducers, and valves do not act actively during sorption experiments. The minimization of these non-active volumes (e.g., by insertion of metallic rods in all tubes [11]) maximizes the difference between amount of *i*-gas in V_A_ at time zero (i.e., immediately after gas is expanded from V_B_ to V_A_) and the same amount at equilibrium; therefore, volume optimization increments the sensitivity of the mixed-gas sorption system and it was crucial to explore the low solubility coefficient of 6FDA-mPDA toward methane at high partial pressures (see later in this work). All volumes of the pressure-decay system were calibrated via a gas expansion procedure (V_A_ = 9.00 ± 0.03 cm^3^; V_B_ = 7.77 ± 0.03 cm^3^; Vc = 10.44 ± 0.04 cm^3^), already well described in the literature [9,10,11,12,13,14], in which a known reservoir volume, which was previously calibrated via water filling at room temperature, is added to V_A_ (or eventually to V_B_). To the best data accuracy, all volume values employed reflected the actual situation of the system—that is, any maintenance-operation on the system was always followed by a leak-test and a re-calibration procedure, and were always very close to the first calibration values.

#### 2.2.2. Barometric Pure-Gas Sorption

CH_4_ and CO_2_ barometric pure-gas sorption experiments were performed in the system shown in Figure 2. A fresh film sample of 1.16 g was loaded and degassed for 24 h at 35 °C. Gas was admitted in V_B_ and was expanded after pressure equilibration to V_A_. Data were acquired continuously at a rate of one point every two seconds using custom-made software operating in LabVIEW (National Instruments™, Austin, USA) until the average pressure variation was approximately −2 × 10^−7^ atm/s during 100 min; above this pressure, variation value uptakes were constant. At equilibrium, more gas was added to V_B_ and expanded to V_A_.

Gas uptakes were estimated as reported previously in the literature [12,13,14,15] from the difference between the amount gas first admitted to V_A_ at time zero and the amount of the same gas not sorbed by the polymer sample at equilibrium. Molar amounts were calculated via the equation of state of gases (corrected with compressibility factor) from pressure transducer readings. The Soave-Redlich-Kwong (S-R-K) equation of state [40,41] was used to estimate compressibility factors (S-R-K compressibility factors matched with values obtained via the virial equation of state [19]) and partial gas fugacities. The S-R-K parameters of CO_2_ and CH_4_ can be found elsewhere [15].

The well-established dual-mode sorption (DMS) model [42] was used to fit CH_4_ and CO_2_ pure-gas uptake data. Because DMS model interpolation of gas uptake from fitted isotherms is usually very accurate, the DMS model was also used for estimation of CO_2_ and CH_4_ solubilities at infinite dilution of 6FDA-mPDA and other polymers discussed in this work (see Supporting Information).

#### 2.2.3. Gravimetric Gas Sorption

Pure-gas sorption experiments were performed via an Intelligent Gravimetric Analyzer (IGA) by Hiden Isochema (Warrington, UK). A fresh sample was loaded in this gravimetric system and degassed at 35 °C under high vacuum (<10^−7^ mbar) for at least 24 h. When the sample weight was stable, sorption measurements were initiated; gas was introduced in the sample chamber at a rate of 0.1 atm/min to reach the desired equilibrium pressure. After equilibration, gas was added cumulatively to obtain a further pressure point.

#### 2.2.4. Barometric Mixed-Gas Sorption and Data Analysis

To perform mixed-gas sorption experiments (see system in Figure 2), the constant feed concentration procedure described in our previous work [11] was applied. In the framework of this procedure, custom-made mixtures were added to V_B_ and, after pressure equilibration, expanded to V_A_. It should be noted that during this expansion procedure, the valve between A and B volumes was opened and then immediately closed to avoid any interaction between the feed gas in V_B_ and the gas of V_A_ now sorbing into the polymer sample (hence, we can assume that at time zero, V_A_ and V_B_ have the same gas composition). At sorption equilibrium (i.e., average pressure variation approximately −2 × 10^−7^ atm/s during 100 minutes), two gas samples from V_B_ and V_A_ were sent to V_C_ for GC analysis. Then, both V_B_ and V_A_ were degassed for a time long enough (about the same time allowed for sorption) to remove any sorbed (detectable) gas from the polymer sample in V_A_; the 6.8 atm range transducer—mounted on V_C_ (see “low range” in Figure 2)—was used to detect gas desorption. If no desorption could be detected, the same gas mixture was added at a higher pressure to V_B_ and then expanded to V_A_ to perform the next mixed-gas uptake experiment. Once an experimental series at fixed mixture composition reached the highest total feed pressure allowed by V_B_ (which depended on the maximum value of gas-cylinder pressure, P_B_ transducer pressure range, and V_B_ dimension), the next experimental series was carried out from a feed pressure of 7 atm. It should be noted that although we set the sequence of the concentration series in the direction of increasing CO_2_ concentrations (i.e., mixtures of higher methane concentration were run first), the sample might have undergone CO_2_-conditioning when going from the last pressure of a series to the first experiment of the new series. This sample conditioning might have introduced a certain over-estimation of a few gas uptake data points; however, we anticipate that this effect was relatively small because between two consecutive experimental data points, the difference in CO_2_ partial pressure was always lower than 8 atm, and because of the extensive sample degassing mentioned above between each experiment. Mixed-gas uptakes were calculated via mass balance and GC composition data [12,13,14,15].

Unless otherwise stated (see Table S4), the same sample used for pure-gas sorption uptakes was employed during mixed-gas sorption experiments.

CO_2_ and CH_4_ mixed-gas sorption uptakes in 6FDA-mPDA in the form of three-dimensional data points were fitted via MATLAB^®^ software (version R2016b, The MathWorks, Inc, Natick, MA, USA). All data were linearly interpolated or were fitted via the DMS model for mixtures [35] supported by an empirical equation for better data fitting. All details of this fitting analysis can be found in the Supporting Information of this work.

#### 2.2.5. Pure- and Mixed-Gas Permeation and Diffusion Coefficients

Pure- and mixed-gas concentration-averaged effective diffusion coefficients for the case of 50:50 mol% CO_2_/CH_4_ feed concentration were calculated as previously done elsewhere [14,24,26,27,43] from ${\bar{D}}_{i}=\;P_{i}/S_{i}^{feed}$; where $P_{i}$ is the pure- or mixed-gas permeability at permeate pressures approaching zero, these permeabilities were previously published by our research group for 6FDA-mPDA [29], and $S_{i}^{feed}$ is the pure- or mixed-gas solubility coefficient of the i-gas at the feed pressure. Because our experimental mixed-gas solubility coefficients were obtained via *constant feed concentration* experiments (i.e., we could only control the concentration at the beginning of the experiment and not at equilibrium [11]), we used models to predict the $S_{i}^{feed}$ values at the fixed 50:50 mol% CO_2_/CH_4_ equilibrium concentration. We predicted these values in two ways: (i) with a modified version of the dual-mode sorption model (details can be found in the Supporting Information), and (ii) with the use of the ‘*linearinterp*’ model within the ‘*fit*’ function of Matlab R2016b. The ‘*linearinterp*’ model simply connects all data points of the 3D uptake diagrams with planes (see Figure S5), therefore, the quality of the prediction of $S_{i}^{feed}$ strictly depends on the number of data points and on experimental accuracy. Both prediction methods for $S_{i}^{feed}$ produced very similar values (see later in the Results and Discussion section).

## 3. Results and Discussion

### 3.1. Experimental Pure- and Mixed-Gas Sorption Data

**Figure 3:**
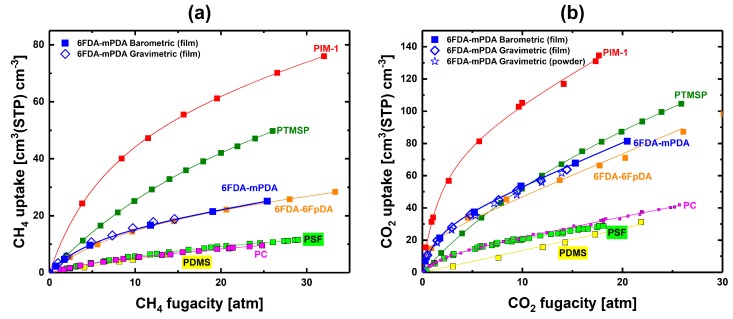
(**a**) CH_4_ and (**b**) CO_2_ pure-gas sorption isotherms at 35 °C vs. gas fugacity. Blue squares were obtained with 6FDA-mPDA films in our custom-built barometric system. Blue diamonds were obtained with 6FDA-mPDA films via gravimetric sorption. CO_2_ pure-gas gravimetric sorption was also performed with a 6FDA-mPDA powder sample (blue stars). Sorption isotherms for polydimethylsiloxane (PDMS) [11], polysulfone (PSF) [45], polycarbonate (PC) [42,46], 6FDA-6FpDA polyimide [44], poly [1-trimethylsilyl-1-propyne] (PTMSP) [15], and polymers of intrinsic microporosity (PIM)-1 [20] are also shown. Interpolations were performed via the dual-mode sorption (DMS) model [42]—DMS parameters were determined in-house. PDMS uptakes were interpolated linearly.

To assess the effects of multicomponent gas sorption on the sorption capacity of the individual mixture components, pure-gas sorption experiments were first performed by barometric and gravimetric techniques. Pure-gas sorption isotherms obtained via the barometric system with a 6FDA-mPDA film were in excellent agreement with those determined gravimetrically (this comparison validated our instrument accuracy) using film and powder samples (Figure 3a,b), indicating that sorption did not depend on the physical state of the 6FDA-mPDA samples.

Figure 3 shows that the 6FDA-mPDA polyimide follows the general pure-gas sorption behavior of another fluorine-containing polyimide, that is, the 6FDA-6FpDA [44] (Figure 3a,b). CO_2_ and CH_4_ isotherms of 6FDA-mPDA are located between the curves of low-free-volume glassy polymers, PSF (polysulfone) and PC (polycarbonate), and high-free-volume PIM-1. The CH_4_ sorption uptake in high-free-volume glassy PTMSP is much higher than in 6FDA-mPDA, as shown in Figure 3a. However, CO_2_ uptakes are comparable for PTMSP and 6FDA-mPDA (Figure 3b). Hence, this example shows how gas/polymer affinity, as well as free volume, plays an important role in gas sorption.

**Figure 4:**
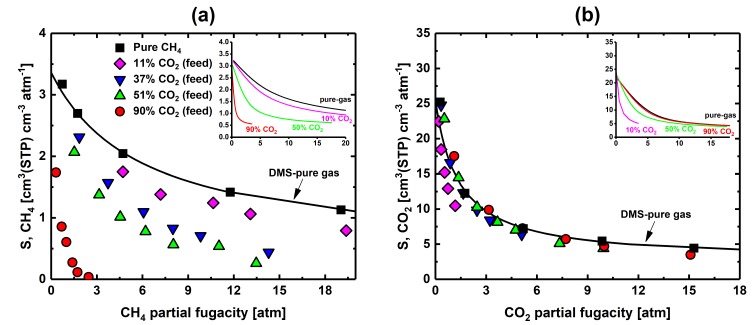
(**a**) Experimental CH_4_ solubility coefficient vs. CH_4_ fugacity and (**b**) CO_2_ solubility coefficient vs. CO_2_ fugacity of 6FDA-mPDA. The inset graphs report the solubility coefficient behavior of CH_4_ and CO_2_ at various equilibrium concentrations—these curves were predicted using the dual-mode sorption model extended to mixtures (DMS-mix) [35] and the pure-gas DMS sorption parameters (Table S1). Note that the feed mixture concentration is the parameter for the experimental data in (**a**,**b**), whereas the concentration at equilibrium is the parameter for the DMS-mix predictions (insert graphs); hence, the comparison between experimental data and predictions is qualitative (i.e., the DMS-mix curves guide the reader through the data).

The CO_2_-CH_4_ mixed-gas solubility coefficient data for 6FDA-mPDA as function of gas fugacity are shown in Figure 4 (all data are also listed in Table S4). The presence of CO_2_ strongly influenced the solubility of CH_4_ (Figure 4a), and, similarly but less markedly, CH_4_ also affected CO_2_ solubility (Figure 4b). The inserts in Figure 4 show the predictions of the extension to mixtures of the dual-mode sorption model (DMS-mix [35]) for CH_4_ and CO_2_, respectively—these inset graphs are intended to provide a reference framework and to guide the reader through the mixed-gas data. The qualitative agreement between predictions of the DMS model and experiments (Figure 4) suggests that competitive sorption in the glassy polymer may be the *main* reason of the deviation of the mixed-gas data from the pure-gas solubility coefficient trends. Other effects that are not accounted for by the DMS model and that impact solubility at high pressures are presented in the Supporting Information of this paper.

Because 6FDA-mPDA has strong affinity to CO_2_, and hence high sorption uptake, the experimental data exhibit small scattering. Conversely, solubility coefficients of methane were scattered at low CH_4_ feed concentrations and at high total pressures, because the accuracy limit of the system was approached (methane solubility coefficients lower than ~0.5 cm^3^(STP) cm^−3^ atm^−1^ require volumes optimization of the barometric pressure decay system, as discussed elsewhere [11]).

### 3.2. Solubility Selectivity Analysis

**Figure 5:**
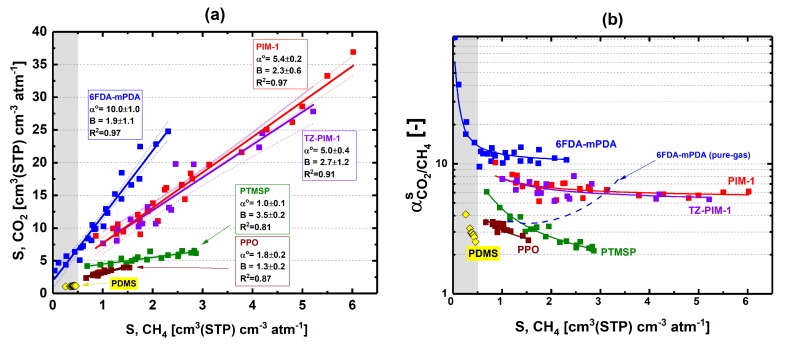
(**a**) CO_2_ vs. CH_4_ mixed-gas solubility coefficient of 6FDA-mPDA at 35 °C—solid lines were estimated via linear fitting of experimental data (the dotted curves delimit the confidence intervals of each linear interpolation); (**b**) data of CO_2_/CH_4_ mixed-gas solubility selectivity vs. CH_4_ mixed-gas solubility coefficient of 6FDA-mPDA (CO_2_/CH_4_ mixed-gas solubility selectivity vs. CH_4_ mixed-gas solubility coefficient mixed- (solid line) and pure-gas trends (dashed-line) are also shown for comparison). Mixed-gas solubility coefficient data from previous reports on PDMS [11], PIM-1 [20], TZ-PIM-1 [23], PTMSP [15], and PPO [18] are included in (**a**,**b**).

We further analyzed our experimental results of mixed-gas solubility for 6FDA-mPDA via a plot of CO_2_ mixed-gas solubility coefficient versus CH_4_ mixed-gas solubility coefficient (Figure 5a). Note that each mixed-gas sorption experiment produces two solubility coefficients: one for CO_2_ and one for CH_4_; hence, these two solubility coefficients generate a single data point in the plot of Figure 5a. Experimental mixed-gas solubility coefficient data at 35 °C are also shown from previous studies for PIM-1 [20], TZ-PIM-1 [23], PTMSP [15], and PPO [18]—data of AO-PIM-1 [23] and polynonene [23] are plotted in Figure S1. Interestingly, all experimental data could be fitted with a straight line regardless of mixture concentration.

The straight line fitted to the data in Figure 5a follows the following equation:

(1) $$S_{CO_{2}}=\alpha_{CO_{2}/CH_{4}}^{o}\cdot S_{CH_{4}}+B$$

where $S_{CO_{2}}$ and $S_{CH_{4}}$ are the solubility coefficient of CO_2_ and CH_4_, respectively; $\alpha_{CO_{2}/CH_{4}}^{o}$ is the mixed-gas selectivity at infinite dilution, that is, the slope of the straight line; and B is the intercept. We rearranged this equation as follows:

(2) $$\alpha_{CO_{2}/CH_{4}}^{mix,S}=\frac{S_{CO_{2}}}{S_{CH_{4}}}=\;\alpha_{CO_{2}/CH_{4}}^{o\;}+\frac{B}{S_{CH_{4}}}$$

where $\alpha_{CO_{2}/CH_{4}}^{mix,\;S}$ is the mixed-gas solubility selectivity coefficient of the membrane material.

When $S_{CH_{4}}$ is high, and $\frac{B}{S_{CH_{4}}}\ll \;\alpha_{CO_{2}/CH_{4}}^{o}$, $\alpha_{CO_{2}/CH_{4}}^{mix,\;S}=\;\alpha_{CO_{2}/CH_{4}}^{o}$ (Equation (2)); this condition may be found for very low equilibrium pressures. Hence, $\alpha_{CO_{2}/CH_{4}}^{o}$ corresponds to the ratio between the solubility coefficient of CO_2_ and CH_4_ at infinite dilution. For most of the polymers shown in Figure 5a, the pure-gas solubility selectivity at infinite dilution (estimated via the DMS model in the form of Equation S2) was in good agreement with the experimental values found via linear interpolation of CO_2_ mixed-gas solubility coefficient versus CH_4_ mixed-gas solubility coefficient data (i.e., $\alpha_{CO_{2}/CH_{4}}^{o}$); this comparison is discussed in the Supporting Information of this work. When $S_{CH_{4}}$ is low (i.e., for high equilibrium pressures), a second limiting condition is found; in this case, the CO_2_/CH_4_ solubility selectivity diverges positively from the $\alpha_{CO_{2}/CH_{4}}^{o}$ value if *B* > 0.

The significance of *B* and $\alpha_{CO_{2}/CH_{4}}^{o}$ in Equations (1) and (2) can be better appreciated in Figure 5b, where the mixed-gas solubility selectivity of 6FDA-mPDA, PIM-1, TZ-PIM-1, PTMSP, PPO, and the predictions of Equation (2) are plotted against the solubility coefficient of CH_4_. For all polymers, predictions by Equation (2) follow the experimental data, and as $S_{CH_{4}}$ decreases, the mixed-gas solubility selectivity increases (*B* > 0)—especially in the grey region of Figure 5. Note that during linear fitting of CO_2_ versus CH_4_ mixed-gas solubilities of 6FDA-mPDA (Figure 5a), the data in the grey region had almost no influence on $\alpha_{CO_{2}/CH_{4}}^{o}$, while they could affect the value of *B*, but not the sign—this confirms the overall trends of solubility selectivity shown in Figure 5b. Interestingly, the mixed-gas data of rubbery PDMS [11] also follow the trend seen for 6FDA-mPDA.

**Figure 6:**
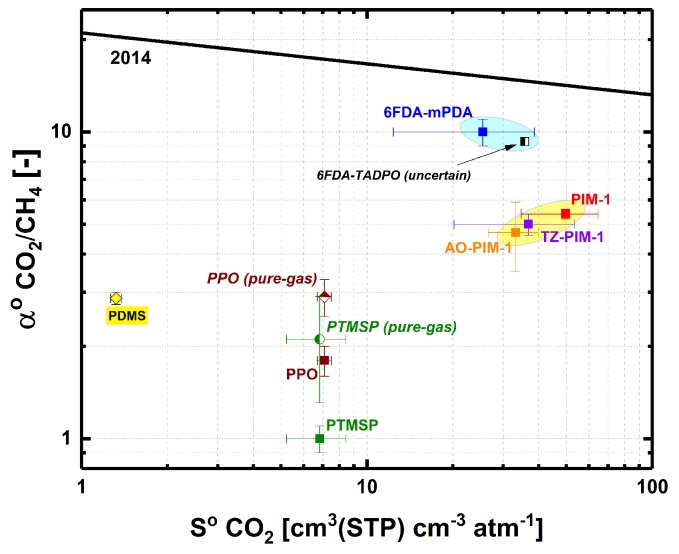
CO_2_ solubility coefficient vs. CO_2_/CH_4_ solubility selectivity (at infinite dilution) data of all polymers tested for CO_2_-CH_4_ mixed-gas sorption at 35 °C [11,15,18,20,22,23,48]. CO_2_/CH_4_ solubility selectivities were obtained from linear interpolation of CO_2_ mixed-gas solubility coefficient vs. CH_4_ mixed-gas solubility coefficient data or from pure-gas uptake data via DMS model equations—see the discussion in the Supporting Information and data values in Table S2. CO_2_ solubility coefficients were estimated from experimental data of pure-gas CO_2_ uptake. The 2014 CO_2_/CH_4_ solubility upper bound was discussed elsewhere [47].

In Figure 5b, the solubility selectivity increases with pressure (*B* > 0) from $\alpha_{CO_{2}/CH_{4}}^{o}$; hence, $\alpha_{CO_{2}/CH_{4}}^{o}$ appears to be a characteristic solubility selectivity value of glassy polymers. We plotted data of $\alpha_{CO_{2}/CH_{4}}^{o}$ versus CO_2_ solubility coefficient at infinite dilution in the 2014 upper bound solubility plot (Figure 6) discussed by Lou et al. [47]. The solubility selectivity at infinite dilution of AO-PIM-1 was obtained from pure-gas uptake experiments (see Supporting Information). For both PTMSP and PPO, the solubility selectivity at infinite dilution estimated from Equation (2) and from the DMS model (i.e., from the ratio of DMS solubility coefficients at infinite dilution calculated via Equation S2) did not agree within the respective standard deviations and were both plotted in Figure 6. The values of $\alpha_{CO_{2}/CH_{4}}^{o}$ and CO_2_ solubility coefficient at infinite dilution for 6FDA-TADPO were uncertain, because of the limited number of pure- and mixed-gas data reported [22,48]. Although the standard error is very large, the pure-gas 6FDA-TADPO solubility trend is qualitatively similar to 6FDA-mPDA (Figure 6). Similarly, PIM-1, TZ-PIM-1, and AO-PIM-1 points group in a confined region of the 2014 CO_2_/CH_4_ solubility upper bound plot.

### 3.3. Equimolar CO_2_-CH_4_ Mixed-Gas Diffusion

**Figure 7:**
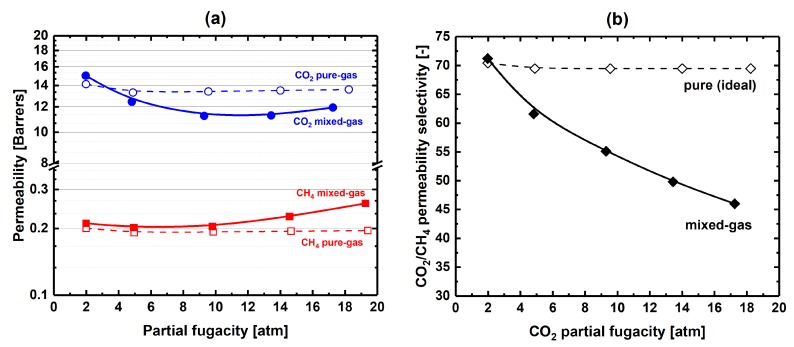
(**a**) CH_4_ and CO_2_ pure- and mixed-gas permeability data (6FDA-mPDA) vs. partial fugacities; (**b**) CO_2_/CH_4_ pure- and mixed-gas permeability selectivity data vs. CO_2_ partial fugacities (feed was equimolar). CH_4_ and CO_2_ permeabilities based on partial pressures were previously reported by our group [29] and corrected with fugacity coefficients. Lines are drawn to guide the eye.

To elucidate the phenomena that affect the separation performance of 6FDA-mPDA polyimide in mixed-gas conditions, we first show previously reported pure- and 50 mol% mixed-gas permeability data of 6FDA-mPDA from our group [29]—here, these data were corrected with fugacity coefficients and re-plotted in Figure 7. Secondly, we incorporate the results of our pure- and mixed-gas solubility experiments to clarify the contribution of mixed-gas solubility to permeability. Finally, CH_4_ and CO_2_ pure- and mixed-gas concentration-averaged effective diffusion coefficients are discussed.

**Figure 8:**
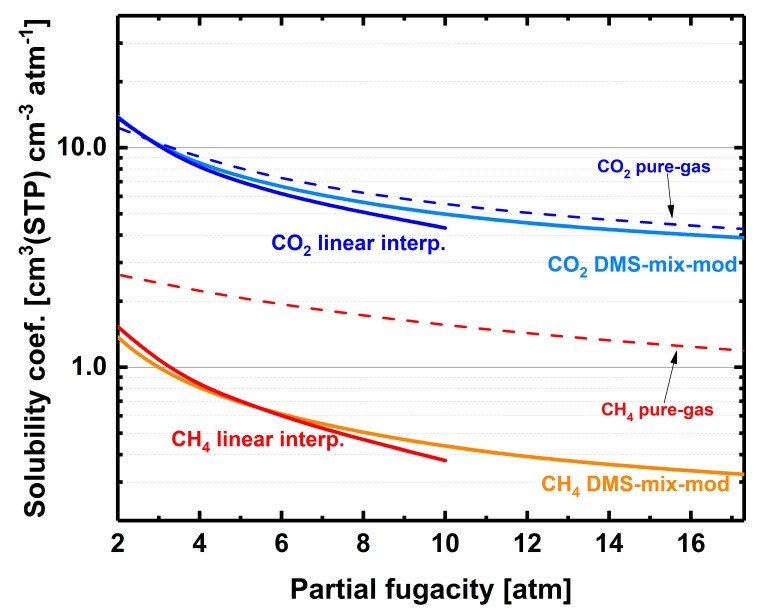
CH_4_ and CO_2_ pure- and mixed-gas solubility coefficients (6FDA-mPDA) vs. partial fugacities. The mixed-gas solubility coefficient curves for methane and carbon dioxide were obtained by linear interpolations of mixed-gas experimental data or by fitting with a modified version of the DMS model for gas mixtures (DMS-mix-mod)—more details can be found in the Supporting Information of this work (Figure S4 and Figure S5).

After correction of partial pressures and driving forces with fugacity coefficients, permeability trends show that CO_2_ mixed-gas permeability suffers from the presence of methane (a local minimum is found at about ~10 atm partial fugacity), whereas CH_4_ mixed-gas permeability increases in mixed-gas conditions, particularly above ~10 atm partial fugacity (Figure 7a). Overall, the permeability selectivity of the mixture strongly diverges from the pure-gas trend (Figure 7b); at about 18 atm partial fugacity, almost 35% of the ideal permeability selectivity is lost. Frequently, it is assumed that competitive sorption is the cause for this loss of permeability selectivity. The data of mixed-gas solubilities at 50 mol% equilibrium concentration (Figure 8) show that competitive sorption strongly affects CH_4_ mixed-gas solubility—that is, when partial pressures increase, the CH_4_ mixed-gas solubility coefficients diverge from the pure-gas values. Because the effect of competitive sorption on the solubility coefficient of CO_2_ is limited, we found that at 2 atm CO_2_ partial pressure, the CO_2_/CH_4_ solubility selectivity jumps from a value of ~5 in the pure-gas state to ~10 in the mixture; moreover, as previously discussed, the CO_2_/CH_4_ mixed-gas solubility selectivity increases with partial pressures. Thus, the effects of gas mixture on solubility selectivity are beneficial during separation of CO_2_ from CH_4_ (equimolar feed) and cannot be held responsible for the loss of permeability selectivity from ideality (Figure 7b).

**Figure 9:**
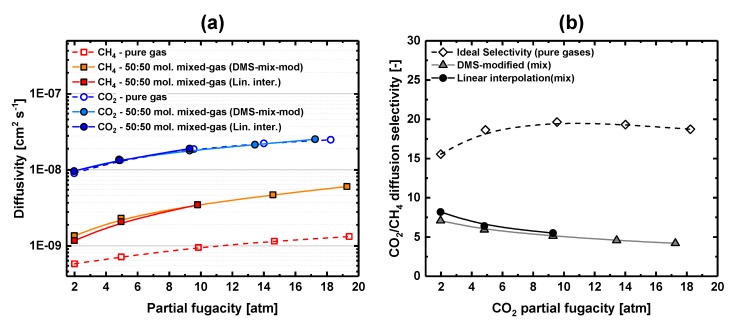
(**a**) CH_4_ and CO_2_ pure- and mixed-gas diffusion coefficients (6FDA-mPDA) vs. partial fugacity; (**b**) CO_2_/CH_4_ pure- and mixed-gas diffusion selectivity vs. CO_2_ partial fugacity. Lines are drawn to guide the eye.

Hence, kinetic effects must be responsible for the deviation of mixed-gas permeability selectivity from the pure-gas values. Figure 9a shows the variation of pure- and mixed-gas concentration-averaged effective diffusion coefficients with partial fugacities. The mixed-gas diffusivities of CH_4_ notably deviate from the pure-gas trend, whereas CO_2_ diffuses in the same manner in pure- and mixed-gas environments with almost no disturbance by methane (Figure 9a). The CO_2_/CH_4_ mixed-gas diffusion selectivity drops from an average pure-gas value of ~18 to a mixed-gas value of ~5 (Figure 9b), simply because in the mixture, CH_4_ diffusion is enhanced compared with that in the pure-gas environment because of the presence of CO_2_ in the mixture. Similar effects on CO_2_-CH_4_ diffusion were observed for PDMS rubber [11]; in this case, sorption of CO_2_ induced the decline of the mixed-gas diffusion and permeability selectivity relative to the pure-gas values. A similar case was discussed by Ribeiro et al. [24], who described how the increase of C_2_H_6_ mixed-gas diffusion coefficient and the decrease of the CO_2_/C_2_H_6_ permeability selectivity could be ascribed to CO_2_-induced “plasticization” of a XLPEO rubber film.

Finally, from the analysis of the data in Figure 7a, one may conclude that “plasticization” of 6FDA-mPDA takes place at partial fugacities greater than ~10 atm, where the gas permeability shows minima for both CO_2_ and CH_4_. However, CH_4_ mixed-gas diffusion rises immediately at low feed pressure after the polymer matrix sorbs CO_2_ (Figure 9a); in other words, the effect of CO_2_ sorption on gas transport occurs over the entire range of partial pressures explored in this work, and the local minima seen in Figure 7a were produced by counteracting thermodynamic and kinetic contributions to transport.

## 4. Conclusions

To quantify the deviation from ideality of CO_2_-CH_4_ mixed-gas permeability and CO_2_/CH_4_ mixed-gas permeability selectivity of 6FDA-mPDA at 35 °C, sorption and diffusion contributions to permeation were decoupled. Experimental data of mixed-gas solubility revealed a decrease of both CO_2_ and, more markedly, CH_4_ solubility due to mixture effects. We found that CO_2_ versus CH_4_ mixed-gas solubility coefficients of 6FDA-mPDA (and other glassy polymers previously studied) follow a linear trend regardless of equilibrium concentration. The slope of the trend line agrees well with the CO_2_/CH_4_ solubility selectivity at infinite dilution, and the intercept indicates the way in which solubility selectivity deviates at increasing pressures. We found the same behavior reviewing mixed-gas sorption data of glassy polymers reported in the literature. In all cases, the CO_2_/CH_4_ solubility selectivity increases with pressure from the value of solubility selectivity at infinite dilution.

Because the CO_2_/CH_4_ solubility selectivity of 6FDA-mPDA improved under mixed-gas conditions, the decline of CO_2_/CH_4_ mixed-gas permeability selectivity from the corresponding pure-gas permeability selectivities—typically observed during CO_2_-CH_4_ permeation in polymeric films—could not be attributed to competitive sorption (as frequently assumed in the literature). Hence, we studied the kinetic behavior of 6FDA-mPDA to elucidate the effect of gas mixture effects on concentration-averaged effective diffusion coefficients as estimated from experimental mixed-gas sorption and permeation data. We observed that after CO_2_ was added to CH_4_ in a mixture, even at a low concentration, the concentration-averaged effective diffusion coefficient of CH_4_ deviated from the pure-gas values, whereas the concentration-averaged effective diffusion coefficient of CO_2_ essentially followed the pure-gas trend; hence, the departure of CO_2_/CH_4_ permeability selectivity of 6FDA-mPDA from the pure-gas values can be explained by a depression of the size sieving capability of 6FDA-mPDA (i.e., it makes CH_4_ diffusion faster than in the pure-gas environment) induced by the presence of CO_2_ by sorption in the polymeric film matrix.

## Supplementary Materials

The following are available online at http://www.mdpi.com/2077-0375/9/1/10/s1. Figure S1: Data of CO_2_ mixed-gas solubility vs. CH_4_ mixed-gas solubility coefficient of AO-PIM-1 and polynonene [2]. For both interpolations, the slope was fixed at the value of pure-gas solubility selectivity at infinite dilution. For both interpolations, the slope was fixed at the value of pure-gas solubility selectivity at infinite dilution. Figure S2: In red and in blue, two examples of the behavior of the *switch* function (Equation S5) used in this work to track the behavior of $K_{D}{}_{i}$ and *b_i_* parameters. In this graph, $A_{i}^{pure}=1$. Figure S3: Comparison between experimental uptakes and model predictions for (a) CH_4_ and (b) CO_2_. Black squares are predictions of the DMS-mix with pure-gas parameters. Red circles are predictions of the DMS-mix-mod. Figure S4: CH_4_ (a) and CO_2_ (b) mixed-gas uptakes in 6FDA-mPDA. Surfaces were obtained via the DMS-mix-mod fitting. The DMS-mix-mod allowed us to predict the solubility behavior beyond the region covered by experimental data. Figure S5: CH_4_ (a) and CO_2_ (b) mixed-gas uptakes in 6FDA-mPDA. Surfaces were obtained via linear interpolation. Table S1: Dual-mode model parameters of methane and carbon dioxide in 6FDA-mPDA for pure-gas sorption at 35 °C. Table S2: Comparison between solubility selectivities at infinite dilution retrieved from mixed-gas and pure-gas sorption data. Table S3: Parameters derived from DMS-mix-mod fitting. Table S4: Pure- and mixed-gas uptake data presented in this work.
