# Supplementary material for: Experimental Mixed-Gas Permeability, Sorption and Diffusion of CO2-CH4 Mixtures in 6FDA-mPDA Polyimide Membrane: Unveiling the Effect of Competitive Sorption on Permeability Selectivity
Source: Membranes (Basel). 2019 Jan 8;9(1):10. doi: 10.3390/membranes9010010 (PMC6359011; doi:10.3390/membranes9010010)
Supplement: Supplementary file 1 [file membranes-09-00010-s001.pdf]

membranes

Supplementary Materials: Experimental Mixed-Gas Permeability, Sorption and Diffusion of CO_2-CH_4 Mixtures in 6FDA-mPDA Polyimide Membrane: Unveiling the Effect of Competitive Sorption on Permeability Selectivity

Giuseppe Genduso, Bader S. Ghanem, Ingo Pinnau

1. Pure-gas DMS parameters and pure-gas solubility coefficient at infinite dilution

The dual-mode sorption (DMS) model for pure-gas uptakes [1] has the following form:

$$C = K_D \cdot f + \frac{C'_H \cdot b \cdot f}{1 + b \cdot f} \qquad \text{S1}$$

where C is the gas sorption uptake, fugacity (f) instead of pressure was used to account for gas non-idealities. In 6FDA-mPDA, CO_2 has higher values of the *Henry's law coefficient* and the Langmuir *holes affinity parameter* (K_D and b, respectively) than methane (Table S1). The Langmuir capacity parameter (C_H') is often used as a measure of the non-equilibrium excess free volume frozen in the glassy polymer.

When $f \to 0$ we obtain the gas solubility coefficient at infinite dilution (Equation S2):

$$S^{\infty} = K_D + C'_H \cdot b \qquad \text{S2}$$

In this work, pure-gas solubility coefficients at infinite dilution could also be estimated via Equation S2 owing to the good quality of the prediction of pure-gas uptakes via the DMS model. In all cases, pure-gas uptake data were digitalized from the literature and fitted with MATLAB® software. In this way, the confidence interval of each parameter could be used to estimate the standard error of pure-gas solubility coefficients and solubility selectivities at infinite dilution.

Table S1. Dual-mode model parameters of methane and carbon dioxide in 6FDA-mPDA for pure-gas sorption at 35 °C.

Gas	K_D (cm³(STP) cm⁻³ atm⁻¹)	C'_H (cm³(STP) cm⁻³)	b (atm⁻¹)	R^2 (-)
**CH_4**	0.44±0.05	17.12±1.82	0.17±0.03	0.999
**CO_2**	2.57±0.32	31.04±6.56	0.74±0.39	0.9995

2. Solubility selectivity analysis

For both AO-PIM-1 and polynonene scattering at low pressure of mixed-gas uptake data (see circled data points Figure S1) limited the quality of the linear fitting of CO_2 mixed-gas solubility coefficient *vs*. CH_4 mixed-gas solubility coefficient data. Data of Figure S1 were fitted by fixing the slope to the value of pure-gas solubility selectivity at infinite dilution. Table S2 compares the values of solubility selectivity at infinite dilution estimated from pure-gas and mixed-gas uptakes of all polymers discussed in this work. 6FDA-mPDA, PIM-1, and TZ-PIM-1 mixed-gas and pure-gas α^{o} values were in agreement. Although the difference was small, in the case of PTMSP and PPO we found a limited agreement. The number of data for 6FDA-TADPO was not enough for reliable linear

fitting of CO_2 *vs.* CH_4 mixed-gas solubility coefficient data; the standard error of both CO_2 pure-gas solubility coefficient and CO_2/CH_4 pure/mixed gas solubility selectivity values at infinite dilution—estimated via the propagation of error theory—were too large (hence, 6FDA-TADPO was not listed in Table S2).

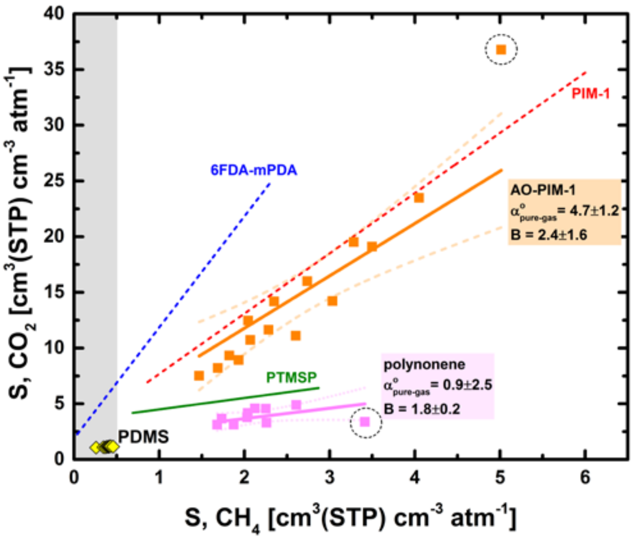

Figure S1. Data of CO_2 experimental mixed-gas solubility coefficient *vs.* CH_4 mixed-gas solubility coefficient of AO-PIM-1 and polynonene [2]. For both interpolations, the slope was fixed at the value of pure-gas solubility selectivity at infinite dilution.

Table S2. Comparison between solubility selectivities at infinite dilution retrieved from mixed-gas and pure-gas sorption data.

Membrane	α^o (pure-gas) (-)	Slope, α^o (mixed-gas) (-)	Intercept, B (mixed-gas) (cm^3(STP) cm^{-3} atm^{-1})	Comment
**6FDA-mPDA [this study]**	7.6±4.1	10.0±1.0	1.9±1.1	α^o, in agreeement
**PIM-1 [3]**	5.5±1.8	5.4±0.2	2.3±0.6	α^o, in agreeement
**TZ-PIM-1 [2]**	5.0±2.5	5.0±0.4	2.7±1.2	α^o, in agreeement
**AO-PIM-1 [2]**	4.7±1.2	-	2.4±1.6	slope fixed to α^o-pure-gas
**PTMSP [4]**	*2.1±0.8*	*1.0±0.1*	*3.8±0.2*	α^o, weak agreement
**PPO [5]**	*2.9±0.4*	*1.8±0.2*	*1.3±0.2*	α^o, weak agreement
**Polynonene [2]**	0.9±2.5	-	1.8±0.2	slope fixed to α^o-pure-gas

3. Fitting/interpolation of mixed-gas sorption data

The extension to a binary mixture of the DMS model (DMS-mix) has the following form:

$$\begin{cases} C_{CH_4} = K_{D\,CH_4} \cdot f_{CH_4} + \dfrac{C'_{H\,CH_4} \cdot b_{CH_4} \cdot f_{CH_4}}{1 + b_{CH_4} \cdot f_{CH_4} + b_{CO_2} \cdot f_{CO_2}} & \text{S3a} \\ C_{CO_2} = K_{D\,CO_2} \cdot f_{CO_2} + \dfrac{C'_{H\,CO_2} \cdot b_{CO_2} \cdot f_{CO_2}}{1 + b_{CO_2} \cdot f_{CO_2} + b_{CH_4} \cdot f_{CH_4}} & \text{S3b} \end{cases}$$

The derivation of this model is discussed elsewhere [6]. The DMS-mix model equations and pure-gas sorption parameters (Table S1) were used to estimate all insert curves plotted in Figure 4a and 4b of the main body of this work.

Solubility coefficients predicted by the DMS-mix have the following relations:

$$\begin{cases} S_{CH_4} = K_{D\,CH_4} + \dfrac{C'_{H\,CH_4} \cdot b_{CH_4}}{1 + b_{CH_4} \cdot f_{CH_4} + b_{CO_2} \cdot f_{CO_2}} & \text{S4a} \\ S_{CO_2} = K_{D\,CO_2} + \dfrac{C'_{H\,CO_2} \cdot b_{CO_2}}{1 + b_{CO_2} \cdot f_{CO_2} + b_{CH_4} \cdot f_{CH_4}} & \text{S4b} \end{cases}$$

When $(1 + b_{CH_4} \cdot f_{CH_4} + b_{CO_2} \cdot f_{CO_2}) >> C'_{H_i} \cdot b_i$ (i.e., at high pressures), CO_2 and CH_4 mixed-gas solubility coefficients and CO_2/CH_4 solubility selectivities converge to the pure-gas values e.g.

$$\alpha_{CO_2/CH_4}{}^{mix} = \alpha_{CO_2/CH_4}{}^{pure} = K_{D\,CO_2}/K_{D\,CH_4}$$

Because we were interested in understanding the effect of polymer/gas1/gas2 interactions during sorption in 6FDA-mPDA, we marked the behavior of K_{D_i} and b_i parameters (DMS-mix) through the following empirical relation:

$$A_i^{mix} = A_i^{pure} \cdot \exp(-\Phi_i\, x_j) \qquad \text{S5}$$

where A could be either K_{D_i} or b_i; x_j is the molar concentration of the *j*-gas (for *i*-*j* mixture) in the sorption atmosphere—for example, to track the deviation of $A_{CO_2}^{mix}$ from $A_{CO_2}^{pure}$ one should vary x_{CH_4}.

When $\Phi < 0$, A_i^{mix} increases with x_j from the pure-gas value (A_i^{pure}) and the opposite happens when $\Phi > 0$—this is also graphically shown in Figure S2.

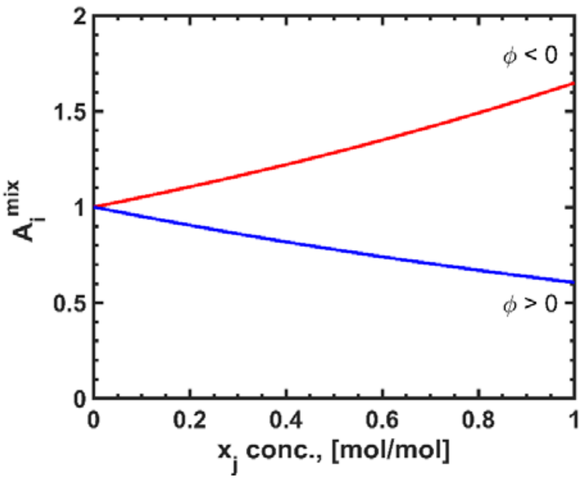

Figure S2. In red and in blue, two examples of the behavior of the *switch* function (Equation S5) used in this work to track the behavior of K_{D_i} and b_i parameters. In this graph, $A_i^{pure} = 1$.

Hence, Equation S5 is used three times in Equation S3a (for $K_{D\,CH_4}$, b_{CH_4}, and b_{CO_2}) and again three times in Equation S3b (for $K_{D\,CO_2}$, b_{CH_4}, and b_{CO_2}). The fitting parameters were $\Phi_{CH_4}^{K_D}$, $\Phi_{CH_4}^{b}$, and $\Phi_{CO_2}^{b}$, for Equation S3a; and, $\Phi_{CO_2}^{K_D}$, $\Phi_{CH_4}^{b}$, and $\Phi_{CO_2}^{b}$, for Equation S3b. We looped the data fitting until $\Phi_{CH_4}^{b}$ of Equation S3a converged with the $\Phi_{CH_4}^{b}$ of Equation S3b—a similar loop was run for $\Phi_{CO_2}^{b}$. In this way, the total number of fitting parameters was four (*i.e.*, $\Phi_{CH_4}^{K_D}$, $\Phi_{CO_2}^{K_D}$, $\Phi_{CH_4}^{b}$ and $\Phi_{CO_2}^{b}$). This data fitting procedure was named DMS-mix-mod.

Table S3. Parameters derived from DMS-mix-mod fitting.

**Gas**	Φ^{K_D} **(-)**	Φ^{b} **(-)**
**CH_4**	1.86	−0.54
**CO_2**	0.34	−0.95

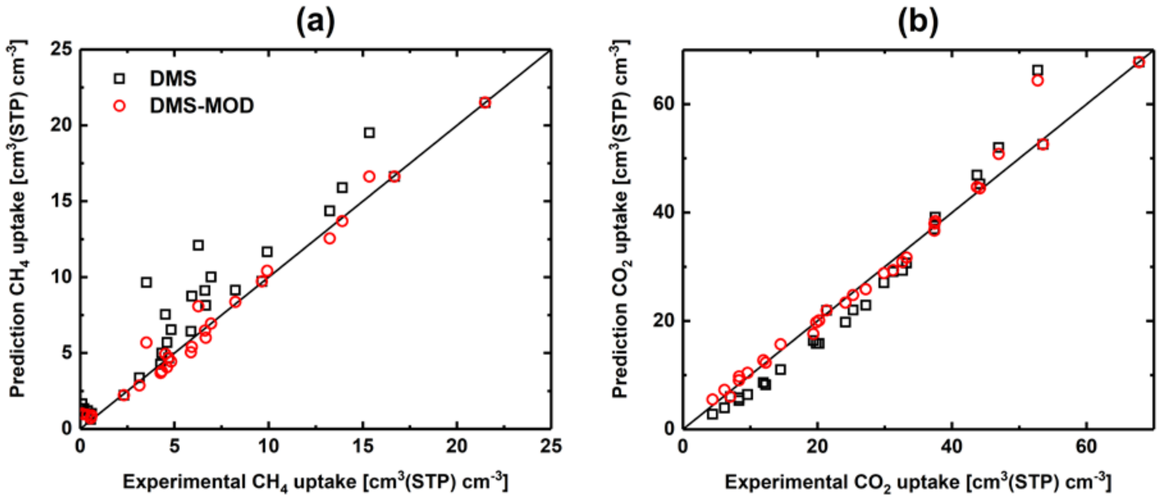

Figure S3. Comparison between experimental uptakes and model predictions for (a) CH_4 and (b) CO_2. Black squares are prediction of the DMS-mix with pure-gas parameters. Red circles are predictions of the DMS-mix-mod.

The result of data fitting via the DMS-mix-mod is shown in Figure S3 and is compared with the prediction of the DMS-mix (Equation 3). Except for some data points, the DMS-mix-mod predicts the experimental data of pure- and mixed-gas uptakes reasonably well. The four parameters that resulted from the fitting analysis through the DMS-mix-mod are listed in Table S3. For K_{D_i}, $\Phi_{CH_4}^{K_D} > \Phi_{CO_2}^{K_D}$ and both are positive; hence—at high pressures—the uptake of both gases negatively deviates from the pure-gas values, whereas the CO_2/CH_4 solubility selectivity is enhanced in the mixture and increases with pressure. This behavior was observed in Figure 5a and Figure 5b of the main body of this paper and coincides with the results of mixed-gas sorption in rubbery membranes discussed elsewhere [7,8]. In the case of b, $\Phi_{CH_4}^{b}$ is slightly higher than $\Phi_{CO_2}^{b}$ and both are negative—the small difference between these two parameters does not allow clear conclusions.

Finally, Figure S4 and Figure S5 show all gas sorption data interpolated via DMS-mix-mod and via linear interpolation, respectively. The curves in red are the curves of 50 mol% concentration at equilibrium. These curves were used to estimate the mixed-gas diffusivity data that are discussed in the main body of this work.

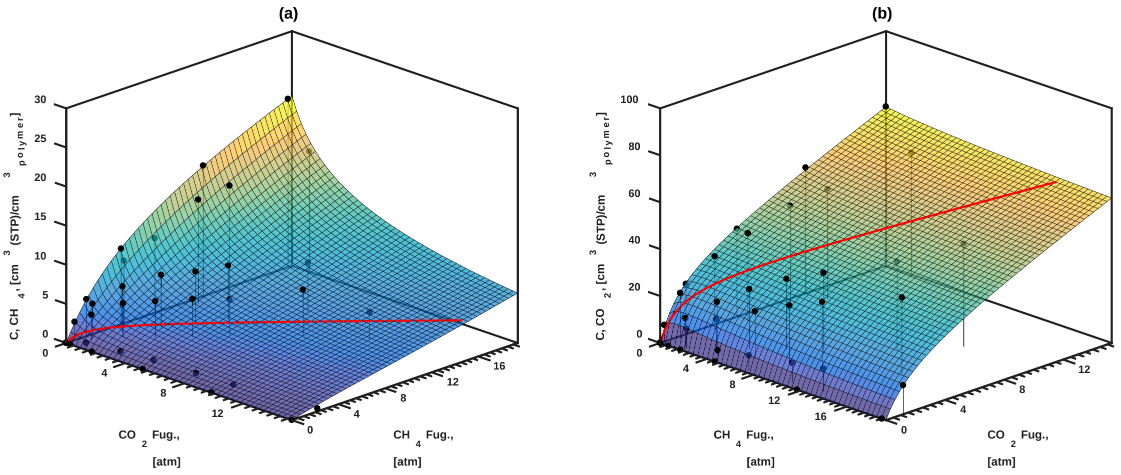

Figure S4. CH_4 (a) and CO_2 (b) mixed-gas uptakes in 6FDA-mPDA. Surfaces were obtained via the DMS-mix-mod fitting. The DMS-mix-mod allowed us to predict the solubility behavior beyond the region covered by experimental data.

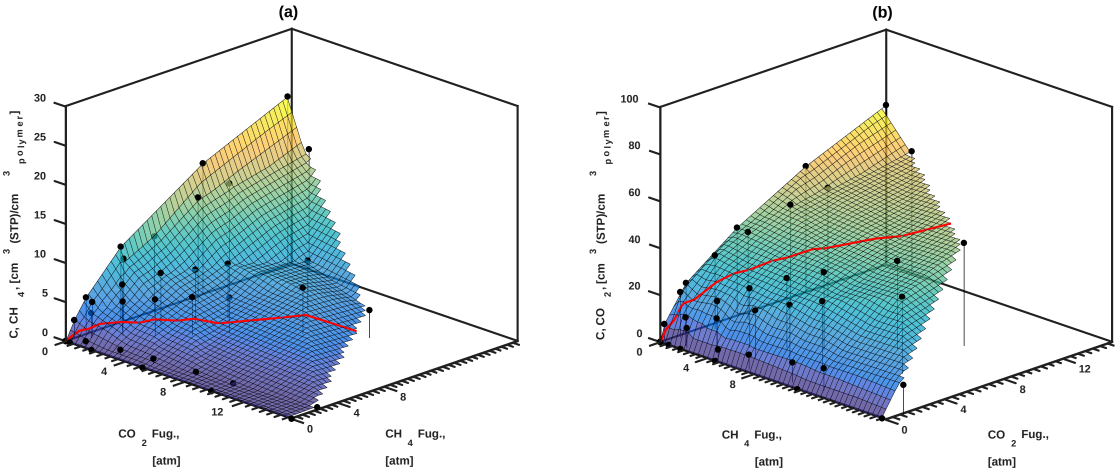

Figure S5. CH_4 (a) and CO_2 (b) mixed-gas uptakes in 6FDA-mPDA. Surfaces were obtained via linear interpolation.

Table S4. Pure- and mixed-gas uptake data presented in this work.

$x_{CH_4}^{feed}$ (mol/mol)	$x_{CH_4}^{equil.}$ (mol/mol)	$f_{CH_4}^{equil.}$ (atm)	$f_{CO_2}^{equil.}$ (atm)	$C_{CH_4}^{equil.}$ (cc(STP)/$cc_{pol.}$)	$C_{CO_2}^{equil.}$ (cc(STP)/$cc_{pol.}$)	
0.00	0.00	0.00	0.28	0.00	7.04	
0.00	0.00	0.00	1.74	0.00	21.35	
0.00	0.00	0.00	5.20	0.00	37.41	
0.00	0.00	0.00	9.85	0.00	53.53	
0.00	0.00	0.00	15.29	0.00	67.82	
0.00	0.00	0.00	20.47	0.00	81.34	
1.00	1.00	0.73	0.00	2.32	0.00	
1.00	1.00	1.74	0.00	4.69	0.00	
1.00	1.00	4.72	0.00	9.64	0.00	
1.00	1.00	11.76	0.00	16.68	0.00	
1.00	1.00	19.04	0.00	21.49	0.00	
1.00	1.00	25.40	0.00	25.13	0.00	
0.89	0.96	4.71	0.20	8.24	4.41	**
0.89	0.95	7.19	0.33	9.92	6.18	**
0.89	0.95	10.65	0.55	13.25	8.34	
0.89	0.94	13.09	0.74	13.91	9.61	
0.89	0.94	19.38	1.17	15.35	12.30	***
0.65	0.84	1.85	0.34	4.27	8.35	
0.64	0.81	3.74	0.88	5.87	14.52	
0.64	0.78	6.07	1.65	6.66	20.24	
0.63	0.76	8.00	2.46	6.62	24.16	
0.63	0.75	9.82	3.23	6.94	27.17	
0.63	0.73	14.29	5.11	6.27	32.62	***
0.50	0.74	1.52	0.52	3.14	11.96	
0.50	0.69	3.15	1.37	4.33	19.82	
0.49	0.64	4.54	2.46	4.60	25.29	
0.49	0.62	6.20	3.67	4.82	29.88	
0.50	0.62	8.03	4.75	4.52	33.25	
0.50	0.59	11.03	7.35	5.93	37.52	
0.48	0.56	13.47	9.93	3.51	43.71	*,***
0.10	0.22	0.32	1.11	0.55	19.40	

0.10	0.18	0.69	3.16	0.59	31.23	
0.10	0.16	1.00	5.15	0.60	37.37	
0.10	0.15	1.40	7.71	0.38	44.14	
0.10	0.14	1.73	9.98	0.20	46.93	
0.10	0.13	2.46	15.09	0.09	52.75	***

Two samples were used during these mixed-gas sorption experiments: sample-1 (1.16 g) used for almost all experiments and sample-2 (0.79 g) used for some repetitions/adding (after completion of the experiments run on sample-1). In particular: * Obtained with sample-2 after checking that CH4 pure-gas uptake agreed with previous observations. ** Tested with both sample-1 and sample-2 (values were averaged). *** Tested with the valve between VB and VC open to increase the feed volume.

References

1. Koros, W.J.; Chan, A.H.; Paul, D.R. Sorption and transport of various gases in polycarbonate. *J. Membr. Sci.* **1977**, *2*, 165–190.
2. Gemeda, A.E. Solubility, diffusivity and permeability of gases in glassy polymers. Alma mater studiorum—Università di Bologna; AMS Dottorato: Bologna, Italy, 2015.
3. Vopička, O.; De Angelis, M.G.; Du, N.; Li, N.; Guiver, M.D.; Sarti, G.C. Mixed gas sorption in glassy polymeric membranes: II. CO_2/CH_4 mixtures in a polymer of intrinsic microporosity (PIM-1). *J. Membr. Sci.* **2014**, *459*, 264–276.
4. Vopička, O.; De Angelis, M.G.; Sarti, G.C. Mixed gas sorption in glassy polymeric membranes: I. CO_2/CH_4 and *n*-C_4/CH_4 mixtures sorption in poly(1-trimethylsilyl-1-propyne) (PTMSP). *J. Membr. Sci.* **2014**, *449*, 97–108.
5. Story, B.J.; Koros, W.J. Sorption of CO_2/CH_4 mixtures in poly(phenylene oxide) and a carboxylated derivative. *J. Appl. Polym. Sci.* **1991**, *42*, 2613–2626.
6. Koros, W.J. Model for sorption of mixed gases in glassy polymers. *J. Polym. Sci.: Polym. Phys. Ed.* **1980**, *18*, 981–992.
7. Genduso, G.; Litwiller, E.; Ma, X.; Zampini, S.; Pinnau, I. Mixed-gas sorption in polymers via a new barometric test system: Sorption and diffusion of CO_2-CH_4 mixtures in polydimethylsiloxane (PDMS). Manuscript submitted for publication.
8. Ribeiro, C.P.; Freeman, B.D.; Paul, D.R. Pure- and mixed-gas carbon dioxide/ethane permeability and diffusivity in a cross-linked poly(ethylene oxide) copolymer. *J. Membr. Sci.* **2011**, *377*, 110–123.
